# Analysis of DNA Polymerases Reveals Specific Genes Expansion in *Leishmania* and *Trypanosoma* spp.

**DOI:** 10.3389/fcimb.2020.570493

**Published:** 2020-10-07

**Authors:** Ana Poveda, Miguel Ángel Méndez, Vinicio Armijos-Jaramillo

**Affiliations:** ^1^DNA Replication and Genome Instability Unit, Grupo de Investigación en Biodiversidad, Zoonosis y Salud Pública (GIBCIZ), Instituto de Investigación en Salud Pública y Zoonosis-CIZ, Facultad de Ciencias Químicas, Universidad Central del Ecuador, Quito, Ecuador; ^2^Departamento de Bioquímica y Biología Molecular, Universidad de Valencia, Burjassot, Spain; ^3^Grupo de Química Computacional y Teórica, Universidad San Francisco de Quito, Quito, Ecuador; ^4^Grupo de Bio-Quimioinformática, Carrera de Ingeniería en Biotecnología, Facultad de Ingeniería y Ciencias Aplicadas, Universidad de Las Américas, Quito, Ecuador

**Keywords:** DNA polymerases, translesion synthesis, trypanosomatids, genome stability, DNA repair, gene amplification

## Abstract

Leishmaniasis and trypanosomiasis are largely neglected diseases prevailing in tropical and subtropical conditions. These are an arthropod-borne zoonosis that affects humans and some animals and is caused by infection with protozoan of the genera *Leishmania* and *Trypanosoma*, respectively. These parasites present high genomic plasticity and are able to adapt themselves to adverse conditions like the attack of host cells or toxicity induced by drug exposure. Different mechanisms allow these adapting responses induced by stress, such as mutation, chromosomal rearrangements, establishment of mosaic ploidies, and gene expansion. Here we describe how a subset of genes encoding for DNA polymerases implied in repairing/translesion (TLS) synthesis are duplicated in some pathogenic species of the Trypanosomatida order and a free-living species from the Bodonida order. These enzymes are both able to repair DNA, but are also error-prone under certain situations. We discuss about the possibility that these enzymes can act as a source of genomic variation promoting adaptation in trypanosomatids.

## Introduction

*Trypanososoma* spp. and *Leishmania* spp. protozoan are the etiological agents responsible of serious parasitosis. These parasites are widely distributed among five continents and represents a global health problem still unsolved (World Health Organization, 2020). Here we highlight the observation of DNA polymerases expansion, that opens a new perspective in terms of evolution/adaptation.

It is well-documented that DNA polymerases are responsible for replicating bulky genomic DNA during the *S* phase, but also have a role to play in repairing/bypassing DNA damage. Therefore, these enzymes play a global role in preserving genetic information and transmitting it to the next generation.

DNA polymerases presents a right-handed structure that embraces the DNA fiber, with three domains: the catalytic palm domain and the most conserved among the polymerases; the thumb domain that is involved in DNA substrate binding and the finger domain that contacts the nascent base pair (Yang and Gao, 2018). Eukaryotic DNA polymerases are classified into four families according to their sequence homology and structural similarities with previous described DNA polymerases: A (*E. coli* Pol I), B (*E. coli* Pol II), X (human Pol β) and Y (*E. coli* UmuC/DinB and eukaryotic *RAD30*) (Steitz, 1999; Burgers et al., 2001). The replicative enzymes, α-primase, pol δ and pol ε (B-family) are multisubunit enzymes. These polymerases are the ones mainly in charge of duplicating DNA during the S-phase and are highly conserved among all the eukaryotes. These polymerases are characterized by their high processivity and accuracy, with a restricted catalytic active site and are only able to accommodate canonical Watson-Crick base paring (A-T, G-C) (Hübscher et al., 2002). However, what often happens is that exogenous and/or endogenous sources induce DNA lesions, which blocks replication fork. In this situation, replicative DNA polymerases are exchanged by the so-called translesion (TLS) DNA polymerases by a tightly regulated cellular pathway (Pillaire et al., 2014; Yang and Gao, 2018).

Once switched, TLS polymerases bypasses DNA lesions, resolving the blocking situations, and they can perform this task in an error-free or error-prone mode. Structurally, TLS are also right-handed but their catalytic center is bigger and less restrictive, being able to accommodate distorted base pairs and bulky lesions and thereby present a lower accuracy (Yang and Gao, 2018). But TLS and other repairing DNA polymerases have also been involved in repairing pathways as BER (Base Excision Repair) or double strand break (DSB) repairing pathways. For more clarity see Supplementary Table 1, the main roles were summarized based on Burgers et al. (2001).

Trypanosomatids encodes also DNA polymerases from A, B, X, and Y families. These were documented in the firsts whole genome sequencing works performed in trypanosomatids (El-Sayed, 2005; Ivens et al., 2005) and annotated in TriTrypDB (Aslett et al., 2010).

## DNA Polymerases Families Overview

**Figure 1:**
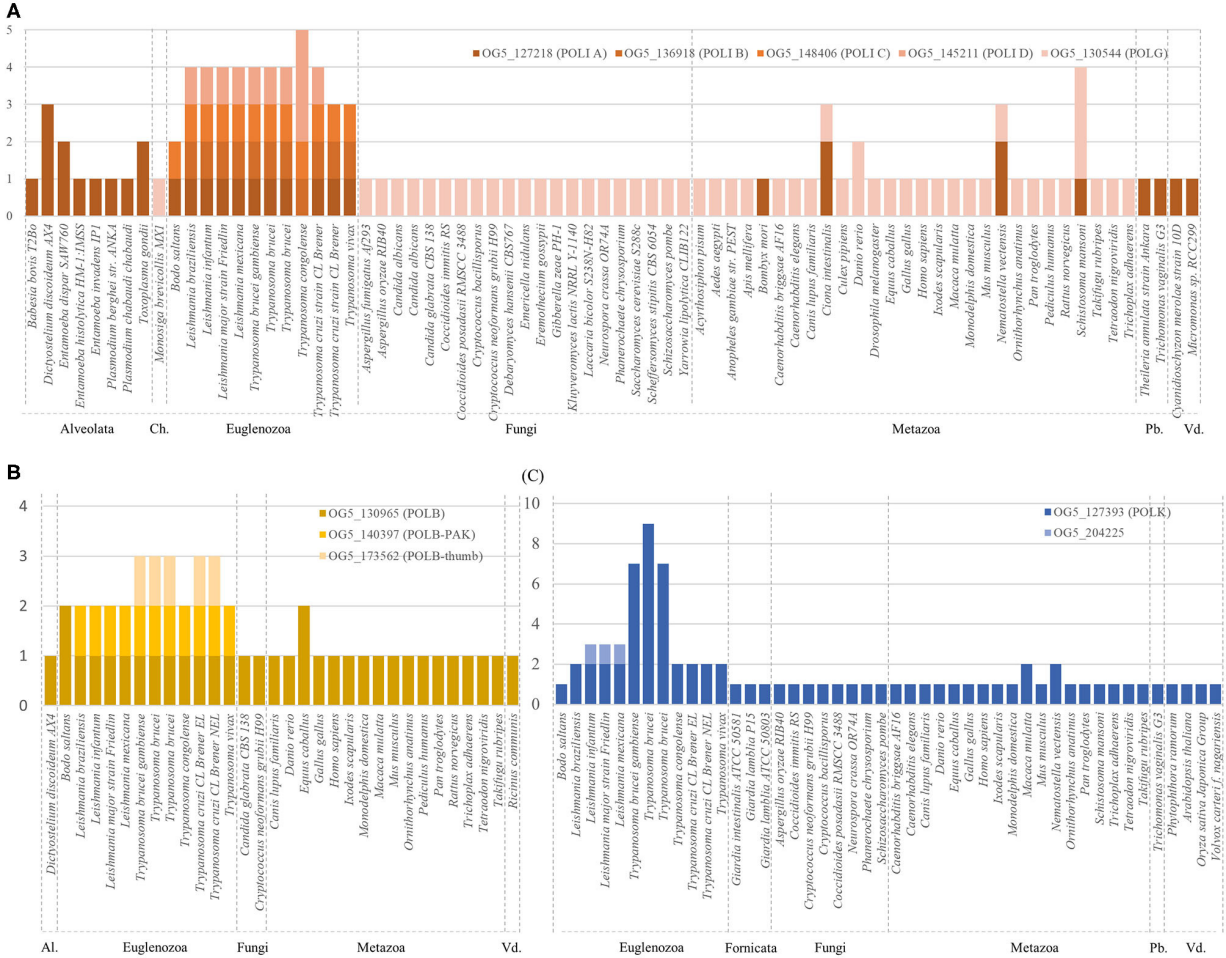
Representation of number of genes (y axe) of DNA polymerases orthologs groups retrieved from OrthoMCL (see Supplementary Table 2). **(A)** Genes related to mitochondrial DNA polymerases (POLI A-D; POLG) from the indicated orthologs groups. **(B)** Genes related to POLB from the indicated orthologs groups. **(C)** Genes related to POLK from the indicated orthologs groups. Species are grouped by *phylum*. ID from represented genes are available at **Supplementary Material**. Ch, Choanoflagellata; Pb, Parabasalids; Vd, Viridiplantae; Al, Alveolata.

Previously we observed that some species of *Trypanosoma* and *Leishmania*, harbors more than one copy of genes encoding for certain DNA polymerases. In order to get a general vision about DNA polymerases genes in species from different *phyla*, orthologs groups were obtained from the OrthoMCL database (Chen et al., 2006) and processed as described in the methodology (Supplementary Table 2). Polymerases with gene duplications in Trypanosomatids were compared with several eukaryotic species (Figure 1). Preliminary results indicate that analyzed kinetoplastids (Euglenozoa *phylum*) species have some non-replicative polymerases duplicated, particularly we will focus on pol β, κ, η, and mitochondrial DNA pol I. Also, some species of different *phyla* seems to harbor more than one copy, but contrarily to Euglenozoa, this is not happening in most of the represented species of the corresponding *phylum*. With the aim of carrying out an in-depth study, we carefully curated the DNA polymerases genes of representative species of the genera *Leishmania* and *Trypanosoma* (Table 1). These parasites are eukaryotes distantly related with fungi and metazoans, and display differences during the DNA replication and repair processes (Uzcanga et al., 2016; da Silva et al., 2018).

**Table 1:** DNA polymerases detected in selected eukaryotic species.

**Family**	***B. saltans***	***T. cruzi***	***T. brucei gambiense***	***L. donovani***	***L. infantum***	***L. mexicana***	***H. sapiens***	***S. cerevisiae***
**A**								
Theta (θ)	BSAL_61675 (helicase domain only) BSAL_89040 (polymerase domain)	TcCLB.509769.70 (helicase domain only) TcCLB.508647.170 (polymerase domain)	Tbg972.8.3110 (helicase domain only) Tbg972.11.6260 (polymerase domain)	LdBPK_231640.1 (helicase domain only) LdBPK_240910.1 (polymerase domain)	LinJ.23.1640 (DEAH helicase domain) LinJ.24.0910 (polymerase domain) (Zhang and Matlashewski, 2019)	LmxM.23.1380 (helicase domain only) LmxM.24.0890 (polymerase domain)	DPOLQ ENSG00000051341	
Gamma (γ)							DPOG1 ENSG00000140521	YOR330C
POLIA	BSAL_50545 (POLIA)	TcCLB.506265.30 (POLIA)	Tbg972.4.2950 (POLIA)	LdBPK_341370.1 (POLIA)	LinJ.34.1370 (Fernández-Orgiler et al., 2016) (POLIA)	LmxM.33.1260 (POLIA)		
POLIB	BSAL_33270 (POLIB)	TcCLB.506227.80 (POLIB)	Tbg972.11.5330 (POLIB)	LdBPK_130080.1 (POLIB)	LinJ.13.0080 (POLIB)	LmxM.13.0080 (POLIB)		
POLIC	BSAL_89100 (POLIC)	TcCLB.506659.20 (POLIC)	Tbg972.7.4480 (POLIC)	LdBPK_140980.1 (POLIC)	LinJ.14.0980 (POLIC)	LmxM.14.0920 (POLIC)		
POLID			Tbg972.11.3630 (POLID)	LdBPK_131370.1.1 (POLID)	LinJ.13.1370 (POLID)	LmxM.13.1630.1 (POLID)		
Nu (ν)							DPOLN ENSG00000130997	
**B**								
Alpha (α)	BSAL_74385	TcCLB.508837.180	Tbg972.8.4690	LdBPK_161640.1.1	LinJ.16.1640	LmxM.16.1540.1	DPOLA ENSG00000101868	YNL102W
Delta (δ)	BSAL_92755	TcCLB.510259.6	Tbg.972.2.350	LdBPK_331790.1.1	LinJ.33.1790	LmxM.32.1690.1	DPOD1 ENSG00000062822	YDL102W
Epsilon (ε)	BSAL_35695	TcCLB.506147.180	Tbg972.9.6060	LdBPK_354430.1.1	LinJ.35.4430	LmxM.34.4360.1	DPOE1 ENSG00000177084	YNL262W
Zeta (ζ)	BSAL_61495 BSAL_61500	TcCLB.509769.130	Tbg972.8.3050	LdBPK_231590.1.1	LinJ.23.1590	LmxM.23.1330.1	REV3L ENSG00000009413	YPL167C
**X**								
Beta (β)	BSAL_83305 nuclear	TcCLB.503955.20 (Schamber-reis et al., 2012; Rojas et al., 2018)	Tbg972.5.3920	LdBPK_080830.1.1	LinJ.08.0830 (Taladriz et al., 2001; Ramiro et al., 2002; Alonso et al., 2006; Khan et al., 2019) Nuclear	LmxM.08.0890.1	DPOLB ENSG00000070501	YCR014C
	BSAL_94795 mitochondrial	TcCLB.503953.59 (PAK-like)	Tbg972.5.3950 (PAK domain)	LdBPK_080840.1.1 (PAK-like)	LinJ.08.0840 (PAK-like)	LmxM.08.0900.1 (PAK-like)		
		TcCLB.506829.30 (thumb domain)	Tbg972.9.2070 (thumb domain) (Saxowsky et al., 2003)					
Lamda (λ)							DPOLL ENSG00000166169	
Mu (μ)							DPOLM ENSG00000122678	
TDT							TDT ENSG00000107447	
**Y**								
Rev1	BSAL_31085	TcCLB.510963.10	Tbg972.10.5510	LdBPK_360110.1.1	LinJ.36.0110	LmxM.36.0100.1	REV1 ENSG00000135945	YOR346W
Eta (η)	BSAL_80970	TcCLB.511911.120	Tbg972.10.2030	LdBPK_210680.1.1	LinJ.21.0680	LmxM.21.0630.1	POLH ENSG00000170734	YDR419W
				LdBPK_210690.1.1	LinJ.21.0690	LmxM.21.0620.1		
Kappa(κ)	BSAL_01685	TcCLB.503755.10	Tbg972.11.9670	LdBPK_281540.1.1	LinJ.28.1540	LmxM.28.1420.1	POLK ENSG00000122008	
		TcCLB.503755.30 (Rajão et al., 2009) mitochondrial	Tbg972.11.9660	LdBPK_281530.1.1	LinJ.28.1530	LmxM.28.1410.1		
			Tbg972.11.9710	LdBPK_281550.1	LinJ.28.1550	LmxM.28.1430.1		
			Tbg972.11.9690					
			Tbg972.11.9680					
			Tbg972.11.9700					
			Tbg972.11.9720					
Iota (ι)							ENSG00000101751	

### Expanded DNA Polymerases Families

The **A-family** of DNA polymerases was described based on sequence homology to DNA pol I (*E. coli*) and Mus 308 (*D. melanogaster*) (Yousefzadeh and Wood, 2013). In metazoans three members have been described belonging to this family: Pol Theta (θ), Pol Nu (ν) and the mitochondrial Pol Gamma (γ). In trypanosomatids, Pol θ and mitochondrial-bacterial PolI related family, are present (see Table 1, Supplementary Figures 1,2).

*Polymerases I (A to D)* belongs to **A-family** and are involved in replicating kinetoplast DNA (kDNA) (Klingbeil et al., 2002; Fernández-Orgiler et al., 2016; Concepción-Acevedo et al., 2018) These genes are phylogenetically distinct from other family. A members, as bacterial DNA PolI (Harada et al., 2020) or the mitochondrial Pol γ of metazoans (Table 1).

kDNA is a complex network of covalently closed minicircles (0.5–2.5 kbp, 5,000 copies) and maxicircles (20–40 kbp, 25 copies) concatenated among them and these protists have developed a unique replication and segregation strategy (Lukeš et al., 2018). In trypanosomes four PolI-like are involved in replicating mitochondrial DNA. In *T. brucei*, PolIB, C, and D are essential for accomplishing the replication of kDNA, while PolIA is not; however, a role in replicating minicircles has been described for PolIB and D (Concepción-Acevedo et al., 2018). We found that *B. saltans* and *T. cruzi* CL Brener Esmeraldo-like lack the PolID, but still have PolI (A-C) coding genes. We observed in TriTrypDB that other *T. cruzi* strains have a copy of PolID, and we do not discard that the lack of this gene in *T. cruzi* CL Brener Esmeraldo-like is a consequence of incomplete sequencing or an annotation failure.

The **X-family** of DNA polymerases in mammals is represented by Pol β, λ, μ, and TdT. In trypanosomatids, the only member present is Pol β (Yang and Gao, 2018). However, between two and three genes can be founded in different species of trypanosomatids. In mammals, X-family polymerases are able to synthesize DNA without a template and play a main role in gap-filling repair after replication in BER, but also in immunoglobulins chain rearrangement (Tdt, Pol μ). The BER pathway is typically implicated in repairing damaged bases resulting from alkylation or oxidation (Yang and Gao, 2018). In humans, researchers have described Pol β mutations associated with poor cancer prognosis, especially after cisplatin treatment (Ray et al., 2013; Nemec et al., 2017).

In trypanosomatids, Pol β is perhaps one of the most studied TLS polymerases. While only one *POLB* gene has been described for *Leishmania* spp (Alonso et al., 2006; Khan et al., 2019), mainly located at the nucleus, in *T. cruzi* (de Oliveira Lopes et al., 2008; Schamber-reis et al., 2012; Rojas et al., 2018) and *T. brucei* (Saxowsky et al., 2003), the enzyme is mainly located in mitochondria. Interestingly, in *T. brucei*, two Pol β variants have been described: Pol β and Pol β-PAK (an Nt domain rich in proline-alanine-lysine, only found in these protozoans). Our analysis shows the presence of Pol β-PAK-like (similar to Pol β-PAK without the presence of PAK domain) in *Leishmania* species and in *T. cruzi*, but not in *B. saltans*. Additionally, we detect the presence of a Pol beta (thumb) in *Trypanosoma* but not in *B. saltans* or *Leishmania* genus (Supplementary Figure 3). We observed that these polymerases share a low sequence similarity (between 7–14%) with the other Pol β studied in this work. Experimental data remains to be carried out to confirm if they are real homologs. In *T. cruzi* has been shown that Pol β and Pol β-PAK-like have dRP lyase activity, and moreover Pol β-PAK-like is able to bypass 7,8-dihydro-8-oxoguanine (8-oxoG), strongly suggesting specialized roles in BER and TLS for kDNA oxidative protection and maintenance (de Oliveira Lopes et al., 2008; Rojas et al., 2018).

In *Leishmania*, the expression and activity of Pol β is regulated at the different biological parasitic stages and responds to external factors such as temperature or pH. Pol β activity is upregulated in intracellular amastigote, located inside an acidic parasitophorous vacuole. In this situation, the parasites should deal with the attack of macrophages that induces oxidative damage. According to this, it has been proposed that Pol β plays a major role in repairing oxidative DNA insults or DNA damage induced by drug exposure (Schamber-reis et al., 2012; Khan et al., 2019). The different sublocalization of Pol β among *Trypanosoma* and *Leishmania* species could be indicative of a different usage of BER at different compartments, a still unsolved question.

The **Y-family** translesion polymerases lack the 3′-5′ exonuclease domain and have a larger active pocket site, able to locate distorted base pairs. These properties make the translesion polymerases error-prone in certain conditions, but certain Y-family polymerases are able to replicate damaged templates with precision. Interestingly, some members of this family are amplified in trypanosomatids (Table 1). In particular, we found the amplification of Pol η and Pol κ genes.

Pol η is present in Opisthokonta- including yeast and vertebrates- and green plants. The *in vivo* function seems to be the TLS replication of specific DNA lesions, mostly in an **accurate** way. Pol η has a predominant role in **accurately** replicating cyclobutane pyrimidine dimer (CPD), the main UV-induced DNA lesion. Actually, humans carrying a mutation in the *POLH* gene develop Xeroderma Pigmentosum type V (XPV), a syndrome characterized for bringing about a predisposition to develop skin cancer through an inability to repair UV-induced damage. In addition, pol η is also able to contribute toward replicating other sorts of lesions: 7,8-dihydro-8-oxoguanine (8-oxoG), one of the most abundant oxidative DNA lesions; and G-G interstrand crosslink, generated for example by cisplatin during chemotherapy, in an error-free manner (Sale, 2013). Overall, it seems that pol η has a protective role against some kinds of lesions, also reported in *T. cruzi* (de Moura et al., 2009). On the other hand, these enzymes are considerably error-prone on undamaged DNA templates and, moreover, an **error-prone** TLS bypass seems to operate in some DNA lesions (for example, 8-nitroguanine (8-nitroG) generated by nitric oxide (NO) metabolism), inducing genomic instability and promoting carcinogenesis (Wu et al., 2006; Kawanishi et al., 2017).

*POLH* genes are duplicated only in the *Leishmania* genus (Table 1, Supplementary Figure 4), with two/three copies in most of them. Remarkably, the copies are located in tandem within the same chromosome. However, *B. saltans* and trypanosomes present only one copy. Based on their sequence, the genes are apparently complete and functional, except for the two copies from *L. donovani* that are truncated. Overall, *POLH* gene duplication seems to be limited to *Leishmania* genus.

However, *POLK* genes amplification is found in *Trypanosoma* spp. and *Leishmania* spp., but not in *B. saltans*. In mammals, Pol κ is the most faithful Y-family polymerase, but is still error-prone. But in general, Pol κ collaborates with Pol ζ to bypass DNA polycyclic aromatic hydrocarbon adducts (such as benzo[a]pyrenes, BDEP), DNA crosslinks (cisplatin), abasic sites and oxidative damage (8-oxo-G or thymine glycol) (Pillaire et al., 2014; Stern et al., 2019; Tonzi and Huang, 2019). So, the efficiency and fidelity of the replication performed depends on the nature of the lesion and the TLS polymerase recruited. Intriguingly, the expression of Pol κ is upregulated in cancerous tumors and it has also been associated with advanced cancer stages (Pillaire et al., 2014).

Pol κ has a Nt domain called N-CLASP, that confers the capacity to encircle the DNA template at the 3'end junction. Additionally, *POLK* genes have two ubiquitin binding zinc finger domains (UBZs) and a Rev1 interacting region (called RIR). All these domains regulate the binding of Pol κ to the replication fork. The RIR domain allows for the interaction of the TLS polymerases κ, η, and ι with the polymerase Rev1. In a previous study that identified Pol κ in trypanosomatids, the authors described the existence of at least two copies of the Pol κ gene in *T. cruzi*, one of them located in the mitochondria (Rajão et al., 2009). This enzyme confers protection against DNA damage and *in vitro* is able to bypass 8-oxoG, indicating a conserved function with respect to other eukaryotes. Also it has been described a role in the extension step on D-loops, an intermediary structure formed during homologous recombination (HR) repairing pathway (Rajão et al., 2009). Similar roles has been described in human Pol η and κ (Sebesta et al., 2013; McVey et al., 2016). However, our BLAST and HMMER searches unveil that *POLK* genes are amplified in trypanosomatids, with 2-3 genes in *Leishmania* spp. and up to seven copies in *T. brucei*. As well as *POLH*, these copies seem to be the result of a duplication process in tandem. Interestingly, only one copy appears in non-parasitic free-living kinetoplastid, *B. saltans* (Table 1).

In our analysis, the κ family shows low sequence conservation (similarity) between paralogs and orthologs. The exception to this observation is *T. brucei gambiense* DAL972 with an expansion in the copy number that is also highly conserved among them (Supplementary Figure 5). This kind of expansion is associated with positive selection to increase the beneficial activity of the duplications (Rogers et al., 2011; Andersson et al., 2015). In order to detect selection over the seven copies of *T. brucei gambiense* DAL972, we performed analyses in Codeml of PAML software and Fixed Effects Likelihood (FEL) of HyPhy. In both cases, sites under positive selection were detected. For Codeml, the M2 model (sites with positive selection) explains the data better than M1 model (sites with relaxation) (*p* = 0.001) using the likelihood-ratio test (LRT). The FEL algorithm also found at least five sites under positive selection with a *p*-value of 0.1. These results suggest a past and/or current adaptive pressure over these sequences.

The duplication of genes involved in DNA repairing and duplication could be a generalized process in trypanosomatids. To test this possibility, we count the number of five genes involved in DNA repairing (RAD1, BRCA2, Ligase 1, and MSH3). We found that these genes are observed in a single copy in the species analyzed here (Supplementary Table 3). The only exception was observed for *T. cruzi* CL Brener Non-Esmeraldo-like with two copies annotated as mismatch repair protein MSH3.

### Discussion

The biological roles of repairing and TLS polymerases in trypanosomatids have been described in recent decades. Here, we want to point out the observation that some families are represented by more than one gene copy, while other polymerases (notably replicative B-family members) present a low fluctuation in number of copies. Here we focus on Pol I, Pol β, and the Y-family Pol η and Pol κ enzymes encoded for more than one allele in trypanosomatids.

#### Life Cycle

The first question one might ask is if in their natural environment, there is a reason why the amplification, selection, -and possible specialization- of these polymerases families have been favored. Once inoculated in the mammal host, trypanosomatids should deal with the host's immune system and macrophage attack to successfully superimpose the infection (Shio et al., 2012; Marr et al., 2014). Two important macrophage responses to the infection are: i) the production of reactive oxygen species (ROS) triggered after phagocytosis; and ii) the production of nitric oxide (NO), generating 8-oxo-G and 8-nitroG, respectively. The noticeable role of Pol η, Pol κ, and Pol β in TLS synthesis and BER repairing, respectively, raises the question of whether the amplification of *POLH/K* and *POLB* genes relies on the need to deal with ROS/NO attacks and overcome DNA lesions.

In trypanosomas Variant Surface Glycoprotein (VSG) antigenic variation is mediated by different strategies involving DNA recombination (McCulloch et al., 2015). Since Y-DNA polymerases are involved in repairing DSB (McIlwraith et al., 2005; Rajão et al., 2009), to delve if they are related in VSG switching is an interesting question.

#### Kinetoplast

Between three and four copies of mitochondrial PolI are present in *Trypanosoma* and *Leishmania* species. kDNA has one of the most complex structures of the eukaryotic mitochondria with a higher amount of DNA in comparison to the nucleus. Indeed, it is a unique structure that provides energy for the cell and for flagella movement. It has been described 3-4 paralogues for DNA Polymerases I in *Trypanosomas* and *Leishmania*. Indeed, *Trypanosomas* have 3-4 orthologs for Pol β and at least one Pol κ in *T. cruzi* is located at the mitochondria. In that sense, the correlation between the number of mitochondrial polymerases and the kDNA size/architecture of the kinetoplast is highly plausible. Some authors have proposed that the increased kDNA size is a neutral evolution triggered by a runaway expansion of DNA (Lukeš et al., 2018). But even if this is true, the cells still need to replicate and ensure the heritage of the uncommon kDNA. So the maintenance of four different enzymes to perform this function is a very likelihood non-neutral evolutionary process.

#### Genome Structure (Gene Copy Number Variation, Polysomic Mosaic)

An interesting characteristic of *Leishmania* is the manifestation of high genomic plasticity. Under different pressure and environmental conditions, different chromosome mosaics are stablished in a few generations, promoting genomic variability (Sterkers et al., 2012; Franssen et al., 2020). On the other hand, evidence involving translesion polymerases in promoting genomic instability and chromosome rearrangements is not fully understood (Kochenova et al., 2011; Sekimoto et al., 2015). But the question here is: are TLS polymerases involved in this process? Of course, further investigation is needed to answer this question.

Interestingly, non-B structured and difficult-to-replicate DNA regions, are present in eukaryotic genomes: Z-DNA, H-DNA triplex, cruciform, hairpins, and G- tetrads (Wickramasinghe et al., 2015; Tsao and Eckert, 2018; Stern et al., 2019). In mammals, Y-family polymerases have been implicated in replicating these particular regions (Eddy et al., 2014, 2016; Wickramasinghe et al., 2015; Quinet et al., 2018; Tsao and Eckert, 2018). Moreover, a role in DNA replication origin has been recently described (Prorok et al., 2019). Trypanosomatids genome have GC-rich sequences and highly repetitive DNA sequences, that organize themselves in no-B structures (Leeder et al., 2016; Belmonte-Reche et al., 2018; Marsico et al., 2019). G-tetrads are present at kinetoplast (Leeder et al., 2016) where a role in RNA editing has been described in the kinetoplast of *T. brucei*. Predictions indicates that G-tetrads are also frequent at telomeres and in less extent at some chromosomes (Belmonte-Reche et al., 2018). Another study points to an enrichment at 5'UTR in trypanosomas (Marsico et al., 2019). The role of polymerases in replicating these G-tetrads and other non-B DNA structures is an interesting issue that remains to be addressed in trypanosomatids.

Overall, different isolated strains present different polysomy and some supernumerary chromosomes, but genome-wide analyses point to the belief that multicopy genes are preferentially located in chromosomes with a normal chromosome copy number (Rogers et al., 2011; Andersson et al., 2015). Remarkably, only pol η and pol κ result from a tandem amplification in all the organism examined, while pol β and pol I genes are distributed among more than one chromosome. It has been proposed that tandem arrays of duplicated genes and/or chromosome ploidies are an alternative way of increasing gene expression, given the particular transcription control mechanism of trypanosomatids. Alternatively, gene amplification can promote specialization. It has been described that some specific gene families are expanded in the *Leishmania* genus (Rogers et al., 2011), many of them related to antigenic surface variation. However, to the best of our knowledge, the expansion of DNA polymerases here described is unusual (Genois et al., 2014; Uzcanga et al., 2016).

### Limitations and Concluding Remarks

Kinetoplastids are eukaryotes that diverged early during the course of evolution, explaining why this class has some exclusive particularities. In recent years, scientists have launched the genome sequencing of several species from different *phyla*, providing lots of new information to the scientific community. Here we point out the observation that some DNA polymerases genes are amplified in species from *Leishmania* and *Trypanosoma* genera, complementing the peculiarities of these organisms.

Intriguingly, this event appears only in gene coding for some specific families, strongly pointing for a particular molecular mechanism with a different evolutionary trajectory. But this work is not a comprehensive study, and cannot provide the mechanisms or advantages that these extra copies can confer in an ecological and/or physiological context. It is plausible to think that these genes can carry out specialized roles, but to describe them further studies are needed.

An attractive question is the possibility that these enzymes can act as a source of genomic variation promoting adaptation in trypanosomatids, given their natural high genomic plasticity. Genomic instability is directly related to evolutionary adaptation (long term) or development of drug resistance (short term). While more experimental data are need to decipher the molecular roles, it would be fascinating to investigate whether and how these extra copies contribute to the high genomic plasticity of trypanosomatids, or not.

### Life Science Identifiers

Life Science Identifiers (LSIDs) for ZOOBANK registered names or nomenclatural acts should be listed in the manuscript before the keywords with the following format:

urn:lsid: < Authority>:<Namespace>:<ObjectID>[:<Version>]

For more information on LSIDs please see Inclusion of Zoological Nomenclature section of the guidelines.

## Author Contributions

AP, MM, and VA-J conceived and planned the study. MM and VA-J performed the bioinformatic analyses. AP and VA-J designed and represented the figures. AP wrote the manuscript. All authors reviewed it.

## Conflict of Interest

The authors declare that the research was conducted in the absence of any commercial or financial relationships that could be construed as a potential conflict of interest.
