# Supplementary material for: Analysis of DNA Polymerases Reveals Specific Genes Expansion in Leishmania and Trypanosoma spp
Source: Front Cell Infect Microbiol. 2020 Oct 7;10:570493. doi: 10.3389/fcimb.2020.570493 (PMC7576959; doi:10.3389/fcimb.2020.570493)
Supplement: Supplementary file 1 [file Table_1.pdf]

Supplementary Material

1 Supplementary Data

The species analyzed in this study were selected while trying to cover human infectious agents from different continents. As a control we chose *B. saltans*, a free-living and non-pathogenic kinetoplastid; and two distant well-studied eukaryotes, namely the yeast *S. cerevisiae* and *H. sapiens*. The *Bodo saltans* strains Lake Konstanz, *Trypanosoma cruzi* CL Brener Esmeraldo-like, *Trypanosoma brucei* gambiense DAL972, *Leishmania donovani* BPK282A1, *Leishmania infantum* JPCM5, and *Leishmania mexicana* MHOM/GT/2001/U1103 proteomes were downloaded from TriTrypDB (Aslett et al., 2010). The proteome of *Homo sapiens* was downloaded from https://www.ensembl.org (Cunningham et al., 2019)and the proteome of *Saccharomyces cerevisiae* ATCC 204508 / S288c from https://www.uniprot.org (UniProt: a worldwide hub of protein knowledge, 2019).

We used the polymerases annotated in *Homo sapiens* as a starting query for the HomoloGene database, NCBI source coordinators 2015. Each homologue group obtained was aligned using MAFFT (default options)(Katoh and Standley, 2013) and then used to create a Hidden Markov models (HMM) with hmmbuild of HMMER 3.1b2 (Eddy and Yang, 2007). We used hmmsearch (using the HMM of each family) to detect polymerases in a database created with the proteomes mentioned above. The results were contrasted with TriTrypDB(Aslett et al., 2010) annotations for the species where the annotation was available.

The homologues identified for each polymerase family were aligned using MAFFT. Then, a phylogenetic reconstruction was performed with PhyML using WAG+G+I model and SH-like as a branch support.

Genes and domains were depicted using DOG 2.0 from The cuckoo Group (Ren et al., 2009). Information for human *POLQ*, *POLG*, *POLB*, *POLH* and *POLK* was retrieved from the bibliography (Despras et al., 2012; Bétous et al., 2013; Belousova and Lavrik, 2015; Lodi et al., 2015; de Lima et al., 2019; Stern et al., 2019).

- To detect signals of positive selection in polymerases, we used Codeml by PAML (Eddy and Yang, 2007). The presence of sites under positive selection were tested by comparing the models M2 (positive selection) and M1 (relaxed selection) using the ETE toolkit 3.0 (Huerta-Cepas et al., 2010). The Likelihood Ratio Test (LRT) was performed ($p \leq 0.05$) to compare the hypotheses. Additionally, we used the Fixed Effects Likelihood (FEL) by HyPhy (Kosakovsky Pond et al., 2020) to detect pervasive selection over sites. The FEL analyses were performed through Datamonkey (Weaver et al., 2018).

Data from Supplementary Table 2 was retrieved from TritrypDB (Aslett et al., 2010) and OrthoMCL (Chen et al., 2006). Data was curated and contrasted with Ensembl.org (Cunningham et al., 2019),

and MetaPhOrs (Chorostecki et al., 2020), NCBI (Coordinators, 2016), Phycocosm and Mycocosm (Nordberg et al., 2014). Filtered data according to the criteria defined at Supplementary Table 2 was used for representations of Figure 1. The *Saccharomyces* genome database (JM et al., 2012), HMMER(Potter et al., 2018), Expasy scanprosite (de Castro et al., 2006), Interpro (Mitchell et al., 2019) and NCBI conserved domain (Lu et al., 2020), Uniprot (UniProt: a worldwide hub of protein knowledge, 2019) and Panther (Mi et al., 2018)were used to annotate and consulting proteins domains.

2 Supplementary Tables

Supplementary Table 1. Main roles of DNA polymerases.

Supplementary Table 2. Excel file with data sheets of DNA polymerase orthologues groups, obtained from OrthoMCL. Orthologues groups' codes are indicated on the top of each sheet.

Supplementary Table 3. Orthologues genes associated with DNA repairing found in TriTrypDB.

Supplementary Table 1. Main roles of DNA polymerases.

Eukaryote DNA polymerases families and main roles	*Human DNA polymerases and functions* (Yang and Gao, 2018)	*Trypanosoma* and *Leishmania* DNA polymerases
A		
DNA repair	Theta (θ); TMEJ- Theta mediated end joining.	Theta (θ)(Fernández-Orgiler et al., 2016; de Lima et al., 2019)
	Nu (ν); end processing.	NA
Mitochondrial DNA replication	Gamma (γ)	NA
	NA	DNA pol I (A-D)* (Klingbeil et al., 2002; Bruhn et al., 2010; Concepción-Acevedo et al., 2018; Harada et al., 2020)
B		
DNA replication	Alpha (α); primer extensión.	Alpha (α); (Leegwater et al., 1991)
	Delta (δ); lagging strand.	Delta (δ)
	Epsilon (ε); leading strand.	Epsilon (ε)
Translesion (TLS) synthesis	Zeta (ζ); TLS extension	Zeta (ζ)
X		
DNA repair	Beta (β); BER-base excision repair; sGRS-small gap filling repair synthesis.	Beta (β); Beta-PAK and Beta-thumb (Taladriz et al., 2001; M. et al., 2002; Saxowsky et al., 2003; Alonso et al., 2006; Lopes et al., 2008; Schamber-reis et al., 2012; Rojas et al., 2018; Khan et al., 2019)

	Lamda (λ); BER-base excision repair; NHEJ-non homologous end joining.	NA
	Mu (μ); NHEJ.	NA
	TDT; NHEJ.	NA
Y		
Translesion (TLS) synthesis	TLS insertion.	Rev1
	TLS insertion.	Eta (η)** (De Moura et al., 2009)
	Kappa (κ); TLS insertion.	Kappa (κ)** (Rajão et al., 2009)
	Iota (ι)	NA

*Between 3 and 4 genes (A-D), depending on the specie.

** Different number of copies, depending on the genus/specie.

Supplementary Table 3. Orthologues genes associated with DNA repairing found in TriTrypDB.

**Gene ID**	**Organism**	**Product Description**	**Ortholog Group**	**Paralog count**
**RAD1**				
BSAL_81005	*B. saltans* strain Lake Konstanz	GPI-anchored surface protein, putative	OG6_147512	0
LINF_200009300	*L. infantum* JPCM5	Cell cycle checkpoint protein RAD1-like – putative	OG6_147512	0
LdBPK_200460.1	*L. donovani* BPK282A1	Cell cycle checkpoint protein RAD1-like, putative	OG6_147512	0
LmxM.20.0390	*L. mexicana* MHOM/GT/2001/U1103	Cell cycle checkpoint protein RAD1-like, putative	OG6_147512	0
Tbg972.1.440	*T. brucei* gambiense DAL972	Cell cycle checkpoint protein RAD1-like, putative	OG6_147512	0
TcCLB.511421.230	*T. cruzi* CL Brener Esmeraldo-like	Cell cycle checkpoint protein RAD1-like, putative	OG6_147512	0
**BRCA2**				
BSAL_00235	*B. saltans* strain Lake Konstanz	BRCA2-like protein, putative	OG6_132920	0
LINF_200005600	*L. infantum* JPCM5	DNA repair protein BRCA2 – putative	OG6_132920	0

LdBPK_200070.1	*L. donovani* BPK282A1	hypothetical protein, conserved	OG6_132920	0
LmxM.20.0060	*L. mexicana* MHOM/GT/2001/U1103	hypothetical protein, conserved	OG6_132920	0
Tbg972.1.100	*T. brucei* gambiense DAL972	hypothetical protein, conserved	OG6_132920	0
TcCLB.505999.40	*T. cruzi* CL Brener Non-Esmeraldo-like	DNA repair protein BRCA2, putative	OG6_132920	0
**Ligase 1**				
BSAL_29980	*B. saltans* strain Lake Konstanz	DNA ligase, putative	OG6_100906	0
LINF_300040100	*L. infantum* JPCM5	DNA ligase I – putative	OG6_100906	0
LdBPK_303490.1	*L. donovani* BPK282A1	DNA ligase I, putative	OG6_100906	0
LmxM.29.3440	*L. mexicana* MHOM/GT/2001/U1103	DNA ligase I, putative	OG6_100906	0
Tbg972.6.4610	*T. brucei* gambiense DAL972	DNA ligase I, putative	OG6_100906	0
TcCLB.506835.120	*T. cruzi* CL Brener Non-Esmeraldo-like	DNA ligase I, putative	OG6_100906	0
**MSH3**				
BSAL_27880	*B. saltans* strain Lake Konstanz	mismatch repair protein MSH3, putative	OG6_105075	0
LINF_150022300	*L. infantum* JPCM5	mismatch repair protein MSH3 – putative	OG6_105075	0
LdBPK_151470.1	*L. donovani* BPK282A1	mismatch repair protein MSH3, putative	OG6_105075	0
LmxM.15.1420	*L. mexicana* MHOM/GT/2001/U1103	mismatch repair protein MSH3, putative	OG6_105075	0
Tbg972.9.2780	*T. brucei* gambiense DAL972	mismatch repair protein MSH3, putative	OG6_105075	0
TcCLB.416511.9	*T. cruzi* CL Brener Non-Esmeraldo-like	mismatch repair protein MSH3, putative	OG6_105075	0
TcCLB.507915.19	*T. cruzi* CL Brener Non-Esmeraldo-like	mismatch repair protein MSH3, putative	OG6_105075	0

For more information on Supplementary Material and for details on the different file types accepted, please see here. Figures, tables, and images will be published under a Creative Commons CC-BY licence and permission must be obtained for use of copyrighted material from other sources (including re-published/adapted/modified/partial figures and images from the internet). It is the

responsibility of the authors to acquire the licenses, to follow any citation instructions requested by third-party rights holders, and cover any supplementary charges.

2.1 Supplementary Figures

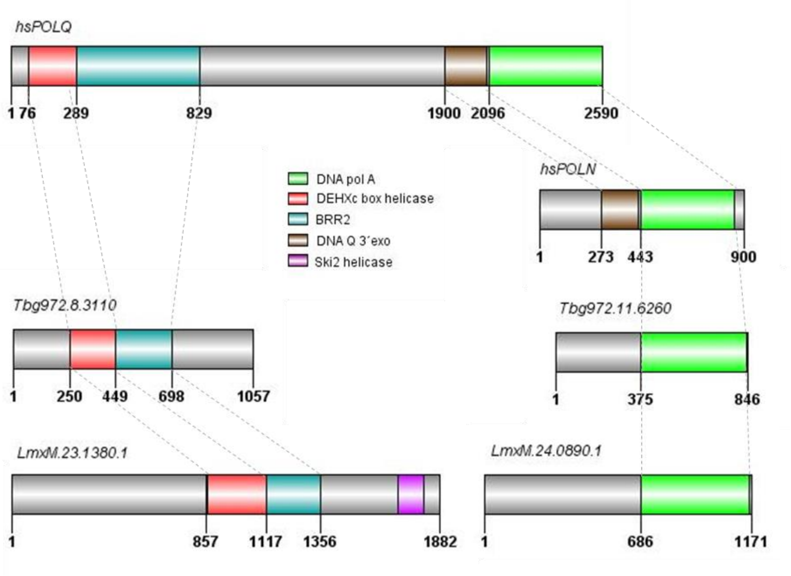

Supplementary Figure 1. Polθ. Scheme of genes and their encoding domains for Polθ from *Homo sapiens* (Hs), *Leishmania mexicana* (LmxM) and *Trypanosoma brucei gambiense* (Tbg972).

A

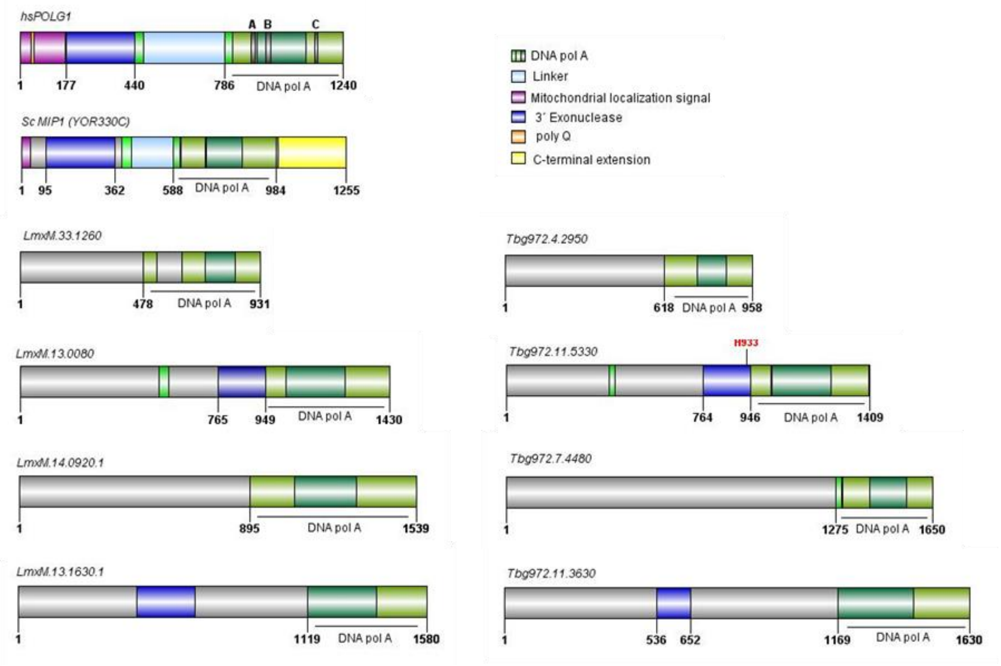

B

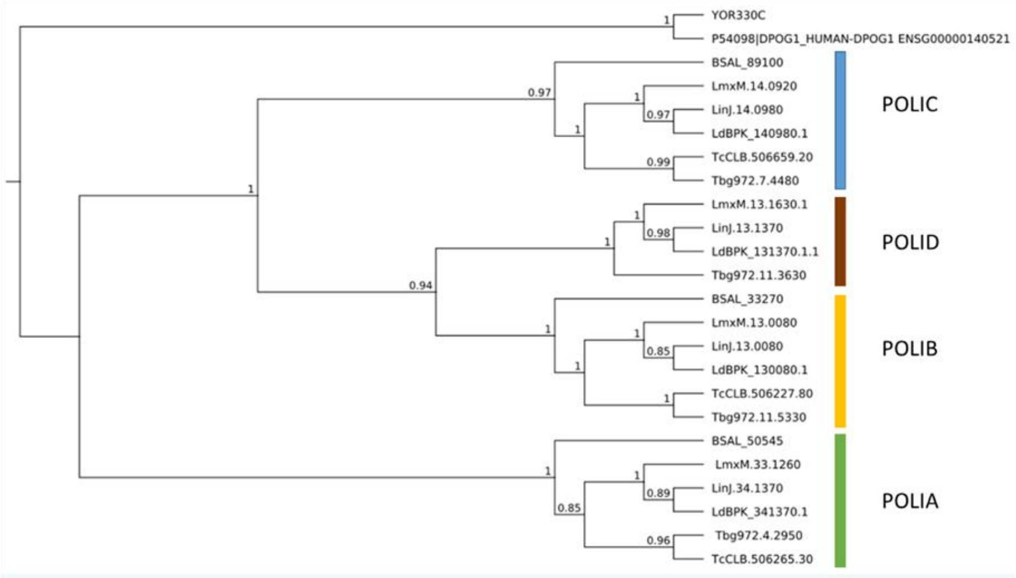

Supplementary Figure 2. PolI. A. Scheme of genes and their domains encoding for Polγ from *Homo sapiens* (Hs), *Saccharomyces cerevisiae* (Sc), and PolI (A-D) for *Leishmania mexicana* (LmxM) and *Trypanosoma brucei gambiense* (Tbg972). B. Phylogenetic tree of PolI. Human and yeast Polγ were used as an outgroup. Clusters of PolI (A-D) were labeled in colors. SH branch support values are presented near to internal nodes of the tree. The genes' IDs were maintained to identify proteins according to the corresponding genome sequence project. The longest predicted protein was used for humans when several isoforms are reported.

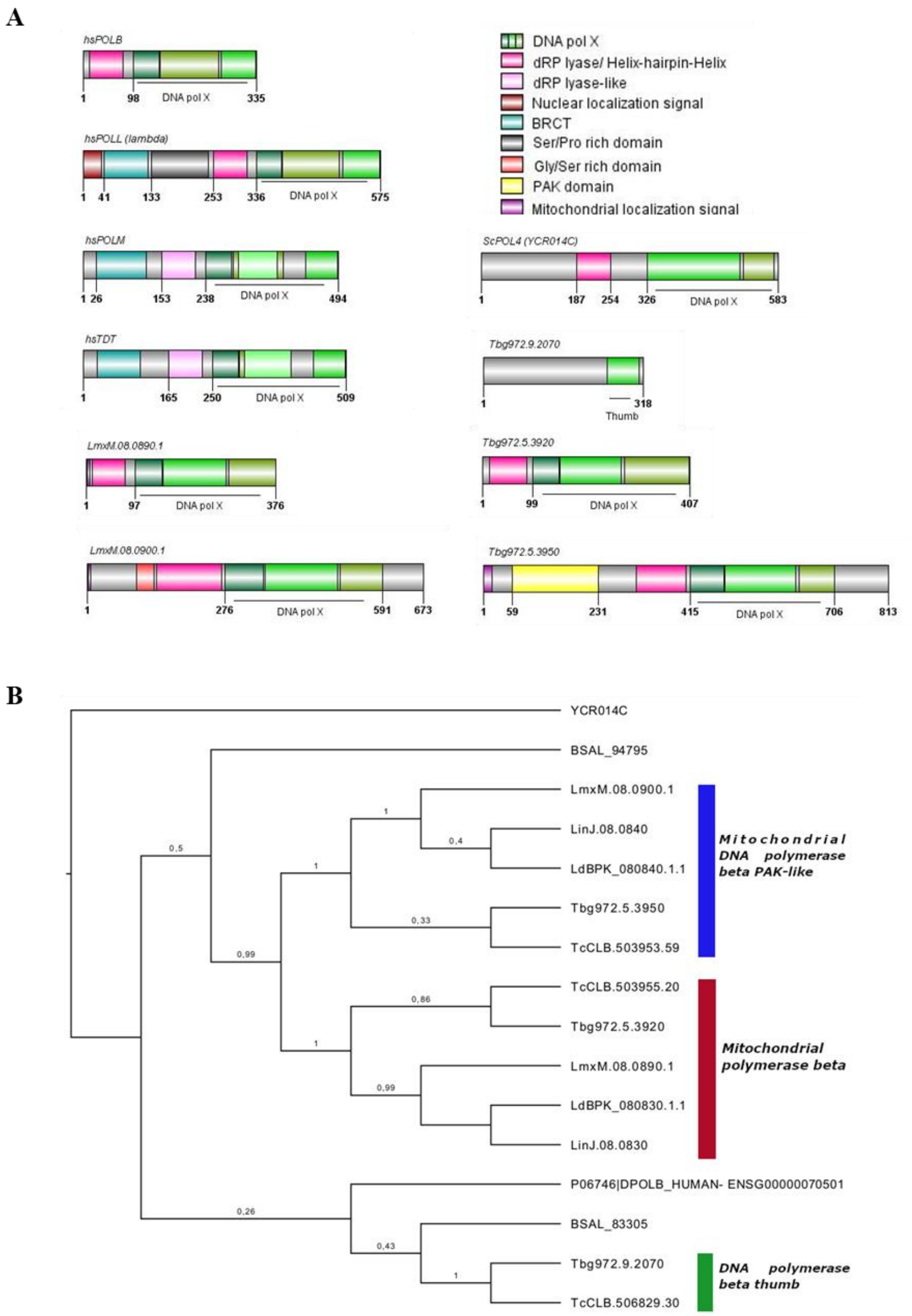

Supplementary Figure 3. DNA polymerase X family. (A) Scheme of genes and their domains encoding for Pol β, λ, μ and TdT from same species described in Supplementary Figure 1. Domains where identified in the bibliography and with the domain databases' researchers (HMMscan, NCBI domain, Expasyprosite and Interproscan). (B) Phylogenetic tree of Polβ. Human and yeast Polβ were used as an outgroup. The groups identified from Polβ are pointed out to the left-hand side of the figure. SH branch support values are presented near to internal nodes of the tree. The genes' IDs were maintained to identify proteins according to the corresponding genome sequence project. The longest predicted protein was used for humans when several isoforms are reported.

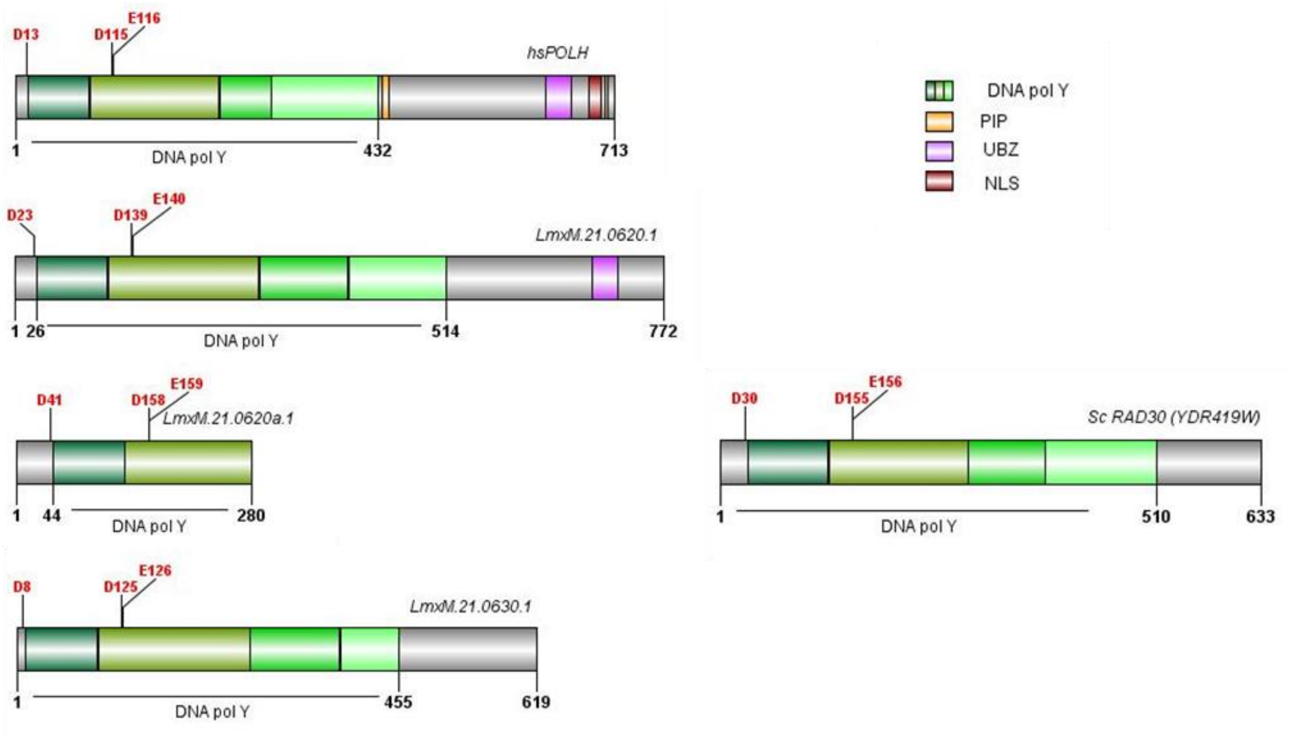

Supplementary Figure 4. Polη Scheme of genes and their domains encoding for Polη from *Homo sapiens* (Hs), *Saccharomyces cerevisiae* (Sc), *Leishmania mexicana* (LmxM) and *Trypanosoma brucei gambiense* strain (Tbg972).

A

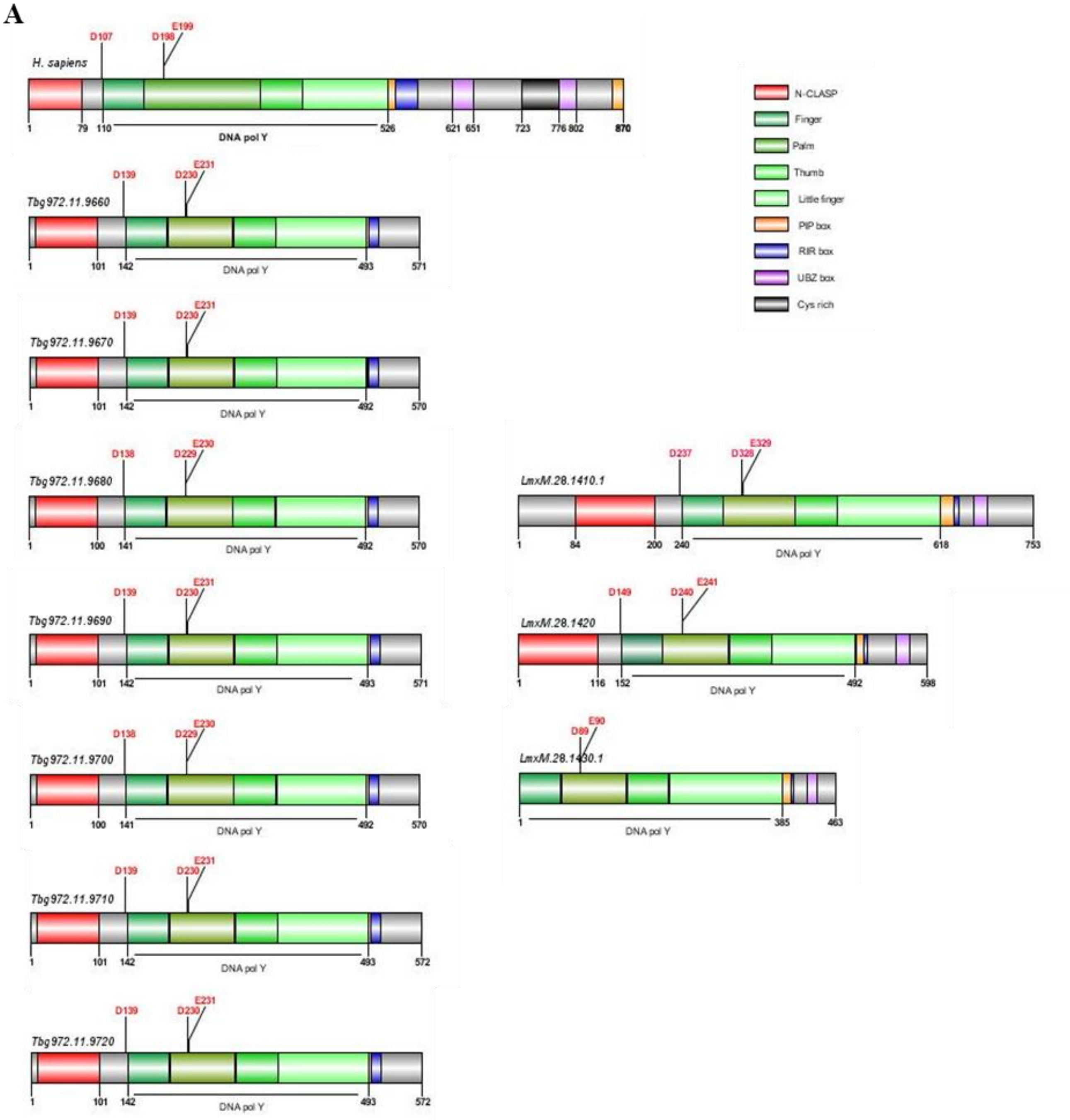
H. sapiens
D107
D198
E199
1
79
110
526
621
651
723
776
802
870
DNA pol Y
N-CLASP
Finger
Palm
Thumb
Little finger
PIP box
RIR box
UBZ box
Cys rich
Tbg972.11.9660
D139
D230
E231
1
101
142
493
571
DNA pol Y
Tbg972.11.9670
D139
D230
E231
1
101
142
492
570
DNA pol Y
Tbg972.11.9680
D138
D229
E230
1
100
141
492
570
DNA pol Y
Tbg972.11.9690
D139
D230
E231
1
101
142
493
571
DNA pol Y
Tbg972.11.9700
D138
D229
E230
1
100
141
492
570
DNA pol Y
Tbg972.11.9710
D139
D230
E231
1
101
142
493
572
DNA pol Y
Tbg972.11.9720
D139
D230
E231
1
101
142
493
572
DNA pol Y
LmxM.28.1410.1
D237
D328
E329
1
84
200
240
618
753
DNA pol Y
LmxM.28.1420
D149
D240
E241
1
116
152
492
598
DNA pol Y
LmxM.28.1430.1
D89
E90
1
385
463
DNA pol Y

B

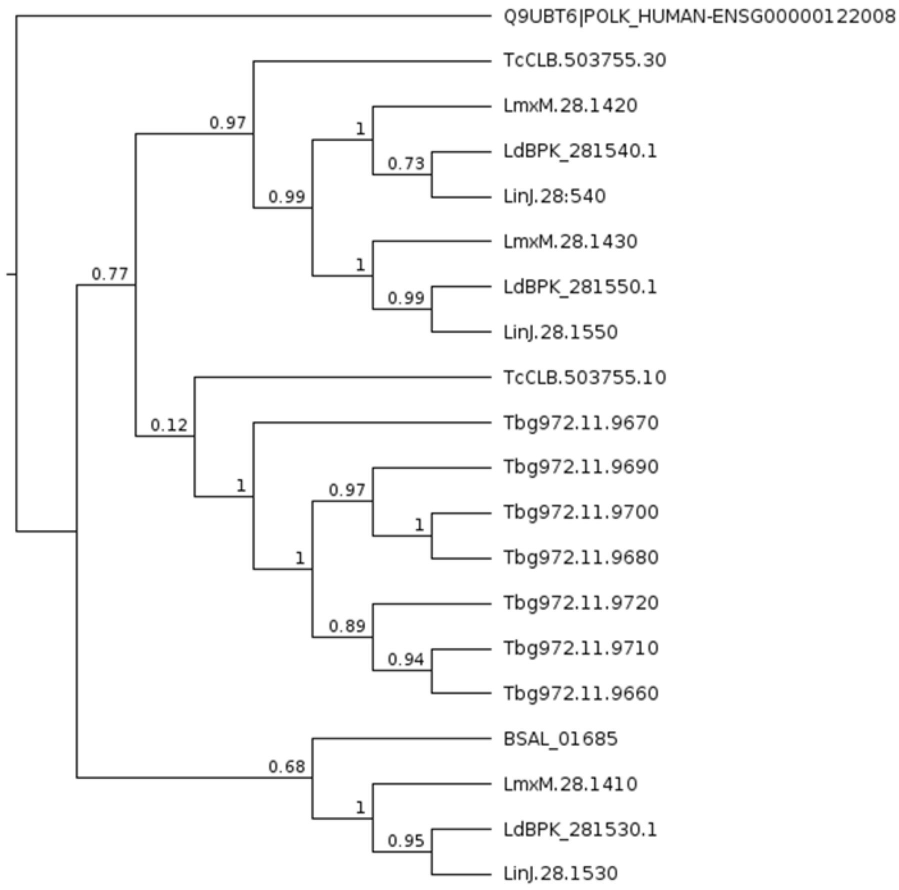

Supplementary Figure 5. Polκ. (A) Scheme of genes and their domains encoding for Polκ from *Homo sapiens* (Hs), *Leishmania mexicana* (LmxM) and *Trypanosoma brucei gambiense* strain (Tbg972) (B) Phylogenetic tree of Polκ obtained from selected species of kinetoplastids. The human Polκ was used as an outgroup. SH branch support values are presented near to internal nodes of the tree. The genes' IDs were maintained to identify proteins according to the corresponding genome sequence project. The longest predicted protein was used for humans when several isoforms are reported.

Alonso, A., Terrados, G., Picher, A. J., Giraldo, R., Blanco, L., and Larraga, V. (2006). An intrinsic 5 -deoxyribose-5-phosphate lyase activity in DNA polymerase beta from Leishmania infantum supports a role in DNA repair. 5, 89–101. doi:10.1016/j.dnarep.2005.08.001.

Aslett, M., Aurrecoechea, C., Berriman, M., Brestelli, J., Brunk, B. P., Carrington, M., et al. (2010). TriTrypDB: a functional genomic resource for the Trypanosomatidae. *Nucleic Acids Res.* 38, D457-462. doi:10.1093/nar/gkp851.

Belousova, E. A., and Lavrik, O. I. (2015). DNA polymerases β and λ and their roles in cell. *DNA Repair (Amst).* 29, 112–126. doi:10.1016/j.dnarep.2015.02.001.

Bétous, R., Pillaire, M.-J., Pierini, L., Laan, S. van der, Recolin, B., Ohl-séguy, E., et al. (2013). DNA polymerase k -dependent DNA synthesis at stalled replication forks is important for CHK1. *EMBO J.* 32, 2172–2185. doi:10.1038/emboj.2013.148.

Bruhn, D. F., Mozeleski, B., Falkin, L., and Klingbeil, M. M. (2010). Mitochondrial DNA polymerase POLIB is essential for minicircle DNA replication in African trypanosomes. *Mol. Microbiol.* 75, 1414–1425. doi:10.1111/j.1365-2958.2010.07061.x.

Chen, F., Mackey, A. J., Stoeckert Jr, C. J., and Roos, D. S. (2006). OrthoMCL-DB: querying a comprehensive multi-species collection of ortholog groups. *Nucleic Acids Res.* 34, D363–D368. doi:10.1093/nar/gkj123.

Chorostecki, U., Molina, M., Pryszcz, L. P., and Gabaldón, T. (2020). MetaPhOrs 2.0: integrative, phylogeny-based inference of orthology and paralogy across the tree of life. *Nucleic Acids Res.* doi:10.1093/nar/gkaa282.

Concepción-Acevedo, J., Miller, J. C., Boucher, M. J., and Klingbeil, M. M. (2018). Cell cycle localization dynamics of mitochondrial DNA polymerase IC in African trypanosomes. *Mol. Biol. Cell* 29, 2540–2552. doi:10.1091/mbc.E18-02-0127.

Coordinators, N. R. (2016). Database resources of the National Center for Biotechnology Information. *Nucleic Acids Res.* 44, D7-19. doi:10.1093/nar/gkv1290.

Cunningham, F., Achuthan, P., Akanni, W., Allen, J., Amode, M. R., Armean, I. M., et al. (2019). Ensembl 2019. *Nucleic Acids Res.* 47, D745–D751. doi:10.1093/nar/gky1113.

de Castro, E., Sigrist, C. J. A., Gattiker, A., Bulliard, V., Langendijk-Genevaux, P. S., Gasteiger, E., et al. (2006). ScanProsite: detection of PROSITE signature matches and ProRule-associated functional and structural residues in proteins. *Nucleic Acids Res.* 34, W362–W365. doi:10.1093/nar/gkl124.

de Lima, L. P., Calderano, S. G., da Silva, M. S., de Araujo, C. B., Vasconcelos, E. J. R., Iwai, L. K., et al. (2019). Ortholog of the polymerase theta helicase domain modulates DNA replication in Trypanosoma cruzi. *Sci. Rep.* 9, 1–16. doi:10.1038/s41598-019-39348-2.

De Moura, M. B., Fonseca Schamber Reis, B. L., Passos-Silva, D. G., Andrade Rajao, M., Macedo, A. M., Franco, G. R., et al. (2009). Cloning and characterization of DNA polymerase eta from Trypano- soma cruzi: roles for translesion bypass of oxidative damage. *Environ. Andmolecularmutagenes.* 50, 375–386.

Despras, E., Delrieu, N., Garandeau, C., Ahmed-Seghir, S., and Kannouche, P. L. (2012). Regulation of the specialized DNA polymerase eta: Revisiting the biological relevance of its PCNA- and ubiquitin-binding motifs. *Environ. Mol. Mutagen.* 53, 752–765. doi:10.1002/em.21741.

Eddy, S. R., and Yang, Z. (2007). PAML 4: phylogenetic analysis by maximum likelihood. *Mol. Biol. Evol.* 24, 1586–1591. doi:10.1093/molbev/msm088.

Fernández-Orgiler, A., MartínezJiménez, I., Alonso, A., Alcolea, P. J., Requena, J. M., Thomas, C., et al. (2016). A putative Leishmania DNA polymerase theta protects the parasite against

oxidative damage. 44, 4855–4870. doi:10.1093/nar/gkw346.

Harada, R., Hirakawa, Y., Yabuki, A., Kashiyama, Y., Maruyama, M., Onuma, R., et al. (2020). Inventory and Evolution of Mitochondrion-localized Family A DNA Polymerases in Euglenozoa. *Pathogens* 9, 257. doi:10.3390/pathogens9040257.

Huerta-Cepas, J., Dopazo, J., and Gabaldón, T. (2010). ETE: a python Environment for Tree Exploration. *BMC Bioinformatics* 11, 24. doi:10.1186/1471-2105-11-24.

JM, C., EL, H., Amundsen, C., Balakrishnan, R., Binkley, G., ET, C., et al. (2012). Saccharomyces Genome Database: the genomics resource of budding yeast. *Nucleic Acids Res.* 40, D700-5.

Katoh, K., and Standley, D. M. (2013). MAFFT multiple sequence alignment software version 7: improvements in performance and usability. *Mol. Biol. Evol.* 30, 772–780. doi:10.1093/molbev/mst010.

Khan, M. I., Mishra, A., Jha, P. K., Abhishek, K., Chaba, R., Das, P., et al. (2019). DNA polymerase β of Leishmania donovani is important for infectivity and it protects the parasite against oxidative damage. *Int. J. Biol. Macromol.* 124, 291–303. doi:10.1016/j.ijbiomac.2018.11.159.

Klingbeil, M. M., Motyka, S. A., and Englund, P. T. (2002). Multiple mitochondrial DNA polymerases in Trypanosoma brucei. *Mol. Cell* 10, 175–186. doi:10.1016/S1097-2765(02)00571-3.

Kosakovsky Pond, S. L., Poon, A. F. Y., Velazquez, R., Weaver, S., Hepler, N. L., Murrell, B., et al. (2020). HyPhy 2.5-A Customizable Platform for Evolutionary Hypothesis Testing Using Phylogenies. *Mol. Biol. Evol.* 37, 295–299. doi:10.1093/molbev/msz197.

Leegwater, P. A. J., Strating, M., Murphy, N. B., Kooy, R. F., Van Der Vliet, P. C., and Overdulve, J. P. (1991). The Trypanosoma brucei DNA polymerase α core subunit gene is developmentally regulated and linked to a constitutively expressed open reading frame. *Nucleic Acids Res.* 19, 6441–6447. doi:10.1093/nar/19.23.6441.

Lodi, T., Dallabona, C., Nolli, C., Goffrini, P., Donnini, C., and Baruffini, E. (2015). DNA polymerase Î³ and disease: what we have learned from yeast. *Front. Genet.* 6. doi:10.3389/fgene.2015.00106.

Lopes, D. de O., Schamber-Reis, B. L. F., Regis-da-Silva, C. G., Rajão, M. A., DaRocha, W. D., Macedo, A. M., et al. (2008). Biochemical studies with DNA polymerase β and DNA polymerase β-PAK of Trypanosoma cruzi suggest the involvement of these proteins in mitochondrial DNA maintenance. *DNA Repair (Amst).* 7, 1882–1892. doi:10.1016/j.dnarep.2008.07.018.

Lu, S., Wang, J., Chitsaz, F., Derbyshire, M. K., Geer, R. C., Gonzales, N. R., et al. (2020). CDD/SPARCLE: the conserved domain database in 2020. *Nucleic Acids Res.* 48, D265–D268. doi:10.1093/nar/gkz991.

M., R., T., H., S., T., and V., L. (2002). DNA polymerase beta mRNA determination by relative quantitative RT-PCR from Leishmania infantum intracellular amastigotes. *Parasitol. Res.* 88, 760–767. doi:10.1007/s00436-002-0653-0.

Mi, H., Muruganujan, A., Ebert, D., Huang, X., and Thomas, P. D. (2018). PANTHER version 14: more genomes, a new PANTHER GO-slim and improvements in enrichment analysis tools. *Nucleic Acids Res.* 47, D419–D426. doi:10.1093/nar/gky1038.

Mitchell, A. L., Attwood, T. K., Babbitt, P. C., Blum, M., Bork, P., Bridge, A., et al. (2019). InterPro in 2019: improving coverage, classification and access to protein sequence annotations. *Nucleic Acids Res.* 47, D351–D360. doi:10.1093/nar/gky1100.

Nordberg, H., Cantor, M., Dusheyko, S., Hua, S., Poliakov, A., Shabalov, I., et al. (2014). The genome portal of the Department of Energy Joint Genome Institute: 2014 updates. *Nucleic Acids Res.* 42, D26-31. doi:10.1093/nar/gkt1069.

Potter, S. C., Luciani, A., Eddy, S. R., Park, Y., Lopez, R., and Finn, R. D. (2018). HMMER web server: 2018 update. *Nucleic Acids Res.* 46, W200–W204. doi:10.1093/nar/gky448.

Rajão, M. A., Darocha, W. D., Franco, G. R., Macedo, A. M., Pena, S. D. J., Teixeira, S. M., et al. (2009). DNA polymerase kappa from Trypanosoma cruzi localizes to the mitochondria , bypasses 8-oxoguanine lesions and performs DNA synthesis in a recombination intermediate. 71, 185–197. doi:10.1111/j.1365-2958.2008.06521.x.

Ren, J., Wen, L., Gao, X., Jin, C., Xue, Y., and Yao, X. (2009). DOG 1.0: illustrator of protein domain structures. *Cell Res.* 19, 271–273. doi:10.1038/cr.2009.6.

Rojas, D. A., Urbina, F., Moreira-Ramos, S., Castillo, C., Kemmerling, U., Lapier, M., et al. (2018). Endogenous overexpression of an active phosphorylated form of DNA polymerase β under oxidative stress in Trypanosoma cruzi. *PLoS Negl. Trop. Dis.* 12, e0006220. doi:10.1371/journal.pntd.0006220.

Saxowsky, T. T., Choudhary, G., Klingbeil, M. M., and Englund, P. T. (2003). Trypanosoma brucei Has Two Distinct Mitochondrial DNA Polymerase β Enzymes. *J. Biol. Chem.* 278, 49095–49101. doi:10.1074/jbc.M308565200.

Schamber-reis, B. L. F., Nardelli, S., Régis-silva, C. G., Carneiro, P., Gonc, P., Almeida, S., et al. (2012). Molecular & Biochemical Parasitology DNA polymerase beta from Trypanosoma cruzi is involved in kinetoplast DNA replication and repair of oxidative lesions. 183, 122–131. doi:10.1016/j.molbiopara.2012.02.007.

Stern, H. R., Sefcikova, J., Chaparro, V. E., and Beuning, P. J. (2019). Mammalian DNA Polymerase Kappa Activity and Specificity. *Molecules* 24, 2805. doi:10.3390/molecules24152805.

Taladriz, S., Hanke, T., Ramiro, M. J., García-Díaz, M., García De Lacoba, M., Blanco, L., et al. (2001). Nuclear DNA polymerase beta from Leishmania infantum. Cloning, molecular analysis and developmental regulation. *Nucleic Acids Res.* 29, 3822–3834. doi:10.1007/s00436-002-0653-0.

UniProt: a worldwide hub of protein knowledge (2019). *Nucleic Acids Res.* 47, D506–D515. doi:10.1093/nar/gky1049.

Weaver, S., Shank, S. D., Spielman, S. J., Li, M., Muse, S. V, and Kosakovsky Pond, S. L. (2018).

Datamonkey 2.0: A Modern Web Application for Characterizing Selective and Other Evolutionary Processes. *Mol. Biol. Evol.* 35, 773–777. doi:10.1093/molbev/msx335.

Yang, W., and Gao, Y. (2018). Translesion and Repair DNA Polymerases : Diverse Structure and Mechanism. *Annu. Rev. Biochem.* 87, 239–261. doi:https://doi.org/10.1146/annurev-biochem-062917-012405.
